# Seizure control by ketogenic diet-associated medium chain fatty acids

**DOI:** 10.1016/j.neuropharm.2012.11.004

**Published:** 2013-06

**Authors:** Pishan Chang, Nicole Terbach, Nick Plant, Philip E. Chen, Matthew C. Walker, Robin S.B. Williams

**Affiliations:** a Centre for Biomedical Sciences, School of Biological Sciences, Royal Holloway University of London, Egham, TW20 0EX, UK; b Centre for Toxicology, Faculty of Health and Medical Sciences, University of Surrey, Guildford, GU2 7XH, UK; c Department of Clinical and Experimental Epilepsy, Institute of Neurology, University College London, WC1N 3BG, UK

**Keywords:** Medium chain triglyceride (MCT) ketogenic diet, Valproic acid (VPA), Seizure control, Teratogenicity, Neuroprotection

## Abstract

The medium chain triglyceride (MCT) ketogenic diet is used extensively for treating refractory childhood epilepsy. This diet increases the plasma levels of medium straight chain fatty acids. A role for these and related fatty acids in seizure control has not been established. We compared the potency of an established epilepsy treatment, Valproate (VPA), with a range of MCT diet-associated fatty acids (and related branched compounds), using *in vitro* seizure and *in vivo* epilepsy models, and assessed side effect potential *in vitro* for one aspect of teratogenicity, for liver toxicology and *in vivo* for sedation, and for a neuroprotective effect. We identify specific medium chain fatty acids (both prescribed in the MCT diet, and related compounds branched on the fourth carbon) that provide significantly enhanced *in vitro* seizure control compared to VPA. The activity of these compounds on seizure control is independent of histone deacetylase inhibitory activity (associated with the teratogenicity of VPA), and does not correlate with liver cell toxicity. *In vivo*, these compounds were more potent in epilepsy control (perforant pathway stimulation induced status epilepticus), showed less sedation and enhanced neuroprotection compared to VPA. Our data therefore implicates medium chain fatty acids in the mechanism of the MCT ketogenic diet, and highlights a related new family of compounds that are more potent than VPA in seizure control with a reduced potential for side effects.

This article is part of the Special Issue entitled ‘New Targets and Approaches to the Treatment of Epilepsy’.

## Highlights

► Medium chain straight and branched fatty acids were examined for seizure control *in vitro.* ► Structural specificity was shown for these compounds. ► Side effects were examined for active seizure-control compounds. ► *In vivo* seizure control, sedation and neuroprotection were shown for these compounds. ► Medium chain fatty acids relating to the MCT ketogenic diet may be active in seizure control.

## 1 Introduction

**Fig. 1:**
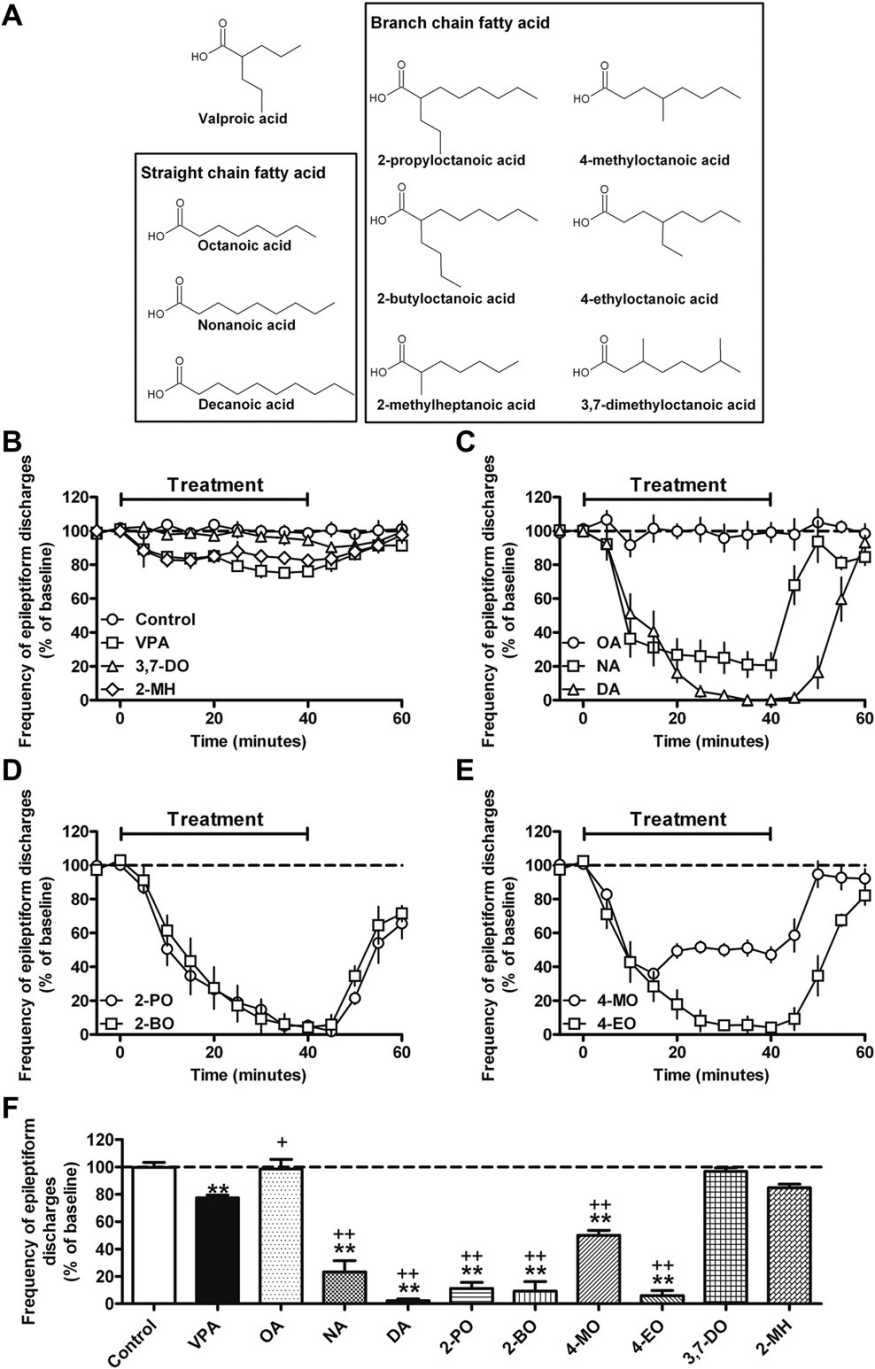
Structurally-specific medium chain fatty acids strongly reduce frequency of *in vitro* epileptiform activity. (A) A range of medium chain fatty acids were analysed in this study: Straight medium chain fatty acids octanoic (OA), nonanoic (NA) and decanoic (DA) acids contain 8, 9 and 10 carbon backbones respectively. Related structures analysed are derivates of octanoic acid branched at the second carbon (2-propyloctanoic acid (2-PO) and 2-butyloctanoic acid (2-BO)) and the fourth carbon (4-methyloctanoic acid (4-MO) and 4-ethyloctanoic acid (4-EO)); and two related structures, a heptanoic acid derivative (7 carbon backbone) branched on the second carbon (2-methylheptanoic acid (2-MH)), and an octanoic acid derivative branched on both the third and the seventh carbon (3,7-dimethyloctanoic acid (3,7-DO)). The frequency of epileptiform activity is plotted against time following (B) control (DMSO) and 3,7-DO (*n* = 3) gave no effect on epileptiform activity, whereas VPA and 2-MH (*n* = 3) treatment showed a weak effect. (C) The straight chain fatty acid OA showed no effect, whereas a strong effect was shown for NA and DA. (D) Octanoic acid derivatives branched on the second carbon, 2-PO, 2-BO; and (E) on the fourth carbon 4-MO, 4-EO are also highly active. (F) Comparison of the mean frequency of PTZ-induced burst discharges, averaged from 20 to 40 min post compound addition (data shown as means ± SEM). * and ** indicate a significant difference at *p* < 0.05 or *p* < 0.01 compared to control respectively; + and ++ indicate similar levels of significance compared to VPA. Data is provided for all compounds tested at 1 mM from at least five repeats unless indicated. Illustrative trace recordings plotted against time for all compounds are provided in Supplementary Fig. 1.

The medium chain triglyceride (MCT) ketogenic diet has provided one of the most effective therapeutic approaches for children with drug resistant epilepsy (Kossoff et al., 2009; Liu, 2008; Neal et al., 2009; Rho and Stafstrom, 2012; Sills et al., 1986b; Vining et al., 1998). However, its use has been limited by poor tolerability, especially in adults, raising the need for the development of novel therapies based upon this diet. The MCT diet causes a rise in ketone body formation, but this correlates poorly with seizure control (Likhodii et al., 2000; Thavendiranathan et al., 2000). It also causes accumulation of medium chain fatty acids in blood plasma (in particular octanoic and decanoic acids, Fig. 1A) (Haidukewych et al., 1982; Newport et al., 1979; Sills et al., 1986a), although the role of these fatty acids, if any, in seizure control remains unclear.

The short chain fatty acid valproic acid (VPA, 2-propylpentanoic acid), is a commonly used broad-spectrum antiepileptic drug, but is sub-optimal due to numerous side effects: The two most significant of these are teratogenicity (Jentink et al., 2010; Koren et al., 2006), which has been correlated with inhibition of histone deacetylase activity (Gottlicher et al., 2001; Gurvich et al., 2004; Phiel et al., 2001) although other mechanism may also function here; and hepatotoxicity (Lagace et al., 2005; Stephens and Levy, 1992), potentially due to effects on β-oxidation (Elphick et al., 2011; Silva et al., 2008). Additionally, fatty acids with structures related to VPA have also been associated with significant sedative properties, often preventing translation into clinical trials (Bojic et al., 1996; Keane et al., 1983; Palaty and Abbott, 1995). These effects have influenced the search for novel fatty acid structures with increased potency against seizures and with a better side-effect profile than VPA.

In the search for new seizure control treatments, a recent study suggested that the action of VPA involves modification of phosphoinositol turnover in the social amoeba *Dictyostelium discoideum* (Chang et al., 2011). Based on this mechanism, a group of medium chain fatty acids including both MCT-diet associated compounds and a novel family of related branched fatty acids were identified as potential new therapeutics for epilepsy. A better understanding of these compounds could therefore aid in the production of more effective treatments for epilepsy.

In this study, we investigated the potency of a range of medium straight and branched chain fatty acids in seizure control using an *in vitro* model system, enabling precise drug dosing, avoidance of confounders such as metabolism/blood brain barrier and rapid throughput, in comparison to VPA. We used an *in vitro* model in which VPA at high doses (2 mM) shows only partial efficacy (Armand et al., 1998) in order to identify compounds that are potentially superior to VPA. We then assessed the resulting active compounds for histone deacetylase inhibition and liver toxicology to identify two promising medium chain fatty acids for further investigation. These two compounds showed dose dependent seizure control in the *in vitro* (PTZ) model, and more potent seizure control than VPA *in vivo*, using a drug-resistant status epilepticus (perforant pathway stimulation model) (Holtkamp et al., 2001). Furthermore, we show that one of these compounds causes less sedation and has a greater neuroprotective (potentially disease modifying) effect than VPA. Our data thus indicate a series of fatty acid which show increased potency in seizure control and potentially reduced side effects compared to the currently available antiepileptic drug, VPA.

## 2 Materials and methods

### 2.1 Animals

Male Sprague-Dawley rats (SD) were kept under controlled environmental conditions (24–25 °C; 50–60% humidity; 12 h light/dark cycle) with free access to food and water. All the experiments were performed in accordance with the guidelines of the Animals (Scientific Procedures) Act 1986. All efforts were made to minimise animal suffering and to reduce the number of animals used.

### 2.2 *In vitro* electrophysiology

The preparation of entorhinal cortex–hippocampus slices and electrophysiological recording in CA1 were described previously (Chang et al., 2010). In brief, SD rats (50–150 g) were decapitated after killing by intraperitoneal injection with an overdose of pentobarbitone (500 mg/kg). The brain was removed and placed in oxygenated ice-cold sucrose solution (in mM: NaCl 87, KCl 2.5, MgCl_2_ 7, CaCl_2_ 0.5, NaH_2_PO_4_ 1.25, NaHCO_3_ 26.2 sucrose 75 and glucose 3). Slices (350 μm) were prepared with a Leica vibratome (Leica VT1200S) and were then stored in an interface chamber containing artificial cerebrospinal fluid solution (aCSF, in mM: NaCl 119, KCl 2.5, MgSO_4_ 1.3, CaCl_2_ 2.5, NaH_2_PO_4_ 1, NaHCO_3_ 26.2 and glucose 16.6) for over 1 h. During the experiment, the slices were transferred from the interface chamber into a submerged recording chamber and continuously perfused with prewarmed (36 °C) oxygenated aCSF (95% O_2_, 5% CO_2_). Field potentials were recorded with a glass microelectrode (1–2 MΩ) filled with aCSF solution placed in stratum radiatum of CA1 and were filtered at 1 kHz and digitized at 2 kHz (using an npi EXT-02F extracellular amplifier recorded with WinEDR software). To induce epileptiform activity, pentylenetetrazol (PTZ) (2 mM) was added to the perfusate and [K^+^] was increased (to 6 mM). Once the frequency of the epileptiform discharges was stable over a period of 10 min, compounds were applied to the perfusate for the following 40 min, and washed out for a remaining 20 min. The anticonvulsant effects were evaluated by measuring the variation of frequency of the discharges every minute. The discharge frequency was then averaged every 5 min during the experiment and normalized to baseline. The testing compounds including VPA (VPA, 0.1, 0.5 and 1 mM) (Sigma), 4-methyloctanoic acid (0.1, 0.5 and 1 mM) (Alfa Aesar) and nonanoic acid (0.1, 0.5 and 1 mM) (Alfa Aesar), octanoic acid (1 mM) (Alfa Aesar), decanoic acid (1 mM) (Alfa Aesar), 4-ethyloctanoic acid (1 mM), 2-propyloctanoic acid (1 mM) (ChemSampCo), 2-butyloctanoic acid (1 mM) (Sigma), 2-methylheptanoic acid (1 mM) (Alfa Aesar) and 3,7 dimethyloctanoic acid (1 mM) (Chemos, Gmbh), and valnoctamide (0.1 mM) (kindly provided by Meir Bialer, The Hebrew University of Jerusalem). Compounds were prepared as 1000 times stocks in dimethyl sulfoxide (DMSO), except VPA was dissolved in distilled water. Stocks were dissolved in aCSF to achieve their final concentrations during experiments.

### 2.3 HDAC activity assay

Histone deacetylase (HDAC) activity was analysed by using an *in vitro* commercial assay, the drug discovery HDAC activity kit (Biomol, Plymouth Meeting, PA). The assays were conducted at room temperature according to the manufacturer's protocol, using Trichostatin A (HDAC Inhibitor; 1 μM) as a positive control. Data was normalized to controls (without VPA or testing compounds) for each experiment.

### 2.4 Hepatic toxicity assay

The Hepatic toxicity assay employed Huh7 cells, kindly provided by Dr Steve Hood (GlaxoSmithKline), cultured in Dulbecco's modified eagle medium (DMEM) with l-glutamine and phenol red, containing 10% foetal bovine serum, 1% non-essential amino acids, 100 U/ml penicillin and 100 μg/ml streptomycin. All cell culture reagents were purchased from Invitrogen (Paisley, UK). Cells were seeded at 1 × 10^4^ cells per well in 96-well plates overnight and grown to approximately 80% cell confluence prior to exposure to 0.01–5 mM testing compound (or vehicle control maintained below 0.5% (v/v) for all conditions). Following exposure to testing compound for 24 h, 0.5 mg/ml 3-(4,5-dimethylthiazol-2-yl)-2,5-diphenyltetrazolium bromide (MTT) was added and cells incubated for a further 2 h to allow colour development. Medium was then aspirated and replaced with 100 μL DMSO per well, and absorbance read at 540 nm.

### 2.5 *In vivo* status epilepticus model

SD rats (300–400 mg) were anesthetized with 1–2% isoflurane in O_2_ (at a flow rate of 2 L/minutes). A recording electrode was stereotaxically implanted into the dentate gyrus (coordinates: lateral 2.5 mm from midline, antero-posterior 4 mm from bregma). A bipolar stimulating electrode was implanted in the right hemisphere and advanced into the angular bundle (coordinates, 4.4 mm lateral and 8.1 mm caudal from bregma) to stimulate the perforant path. These stereotaxic coordinates relative to bregma were based on the atlas of Paxinos and Watson (1998). The depths of the electrodes were adjusted to maximize the slope of the dentate granule cell field potential. Following two weeks recovery, the perforant path was electrically stimulated with 3–5 mA 50 μsec monopolar pulses at 20 Hz for 2 h to induce self-sustaining status epilepticus (SSSE), and the field potential was recorded from the dentate granule bandpass-filtered (0.1–50 Hz), and then digitized at 100 Hz using a CED micro 1401 and SPIKE 2 software (both CED, Cambridge, United Kingdom). After 10 min of SSSE, testing compounds (VPA, 4-methyloctanoic acid or nonanoic acid; 400 mg/kg) or vehicle control (DMSO) were administered intraperitoneally (i.p.), and behavioural seizures and EEG monitored for 3 h. At this point diazepam (10 mg/kg, i.p.) was administered to all animals to stop the SSSE. If necessary, this dose of diazepam was repeated until motor and EEG seizure activity ceased in all animals. Behavioural seizures were monitored every 10 min and classified using the Racine scale. Stage 1, facial automatisms; Stage 2: head nodding and more severe facial and mouth movements; Stage 3: forelimb clonus with a lordotic posture; Stage 4: bilateral forelimb clonus continues along with rearing; Stage 5: falling to one side first and forelimb clonus.

The *in vivo* data were analysed by repeated ANOVA analysis with time of measurement as the within-subject factor and the treatment as the between-subject factor. Post-hoc analysis of treatments against control was performed using 2-sided Dunnett *t* test and comparison between treatments was performed using Tukey HSD test in SPSS (version 20).

For histology, animals were sacrificed 2 months post-SSSE, with an overdose of pentobarbital sodium (500 mg/kg i.p.) (Fort dodge Animal Health, Southampton, UK) and the brains were immersed in 4% paraformaldehyde in 0.1 M phosphate-buffered saline pH 7.4 (PBS) for over 24 h and then stored at 4 °C prior to transfer to PBS with 30% sucrose as a post-fixation solution. Brain sections (40 μm thick thickness) were cut using a vibroslicer (Leica VT 1000S Microsystems, Wetzlar, Germany) and Nissl staining was used to confirm the position of electrodes and examine the degree of neurodegeneration in the hilus using particle analysis in ImageJ software (this calculates the proportion of the hilus in which cell nuclei are present) in comparable sections from each animal (averaged data from four slices per treatment).

### 2.6 Sedation experiment

VPA, 4-methyloctanoic acid and nonanoic acid were administered (i.p.) at 200, 400 or 600 mg/kg to male SD rats. The degree of sedation was assessed by using a well-established scale: 0, spontaneous movement; 1, intermittent spontaneous movement; 2, no spontaneous movement; 3, loss of auditory reflex; 4, loss of corneal reflex; 5, loss of response to tail pinch (Fisher et al., 2004; Holtkamp et al., 2001; Lee et al., 1998). The level of sedation was recorded at 10 min after i.p. injection, and thereafter every 30 min for 3 h.

### 2.7 Statistical analysis

In all data provided, results are presented as mean ± SEM. Statistical comparisons were performed by using the Mann–Whitney test, and one way ANOVA followed by Dunnett for post hoc analysis using SPSS.

## 3 Results

### 3.1 The effect of medium chain fatty acids on *in vitro* seizure control activity

**Supplementary Fig. 1:**
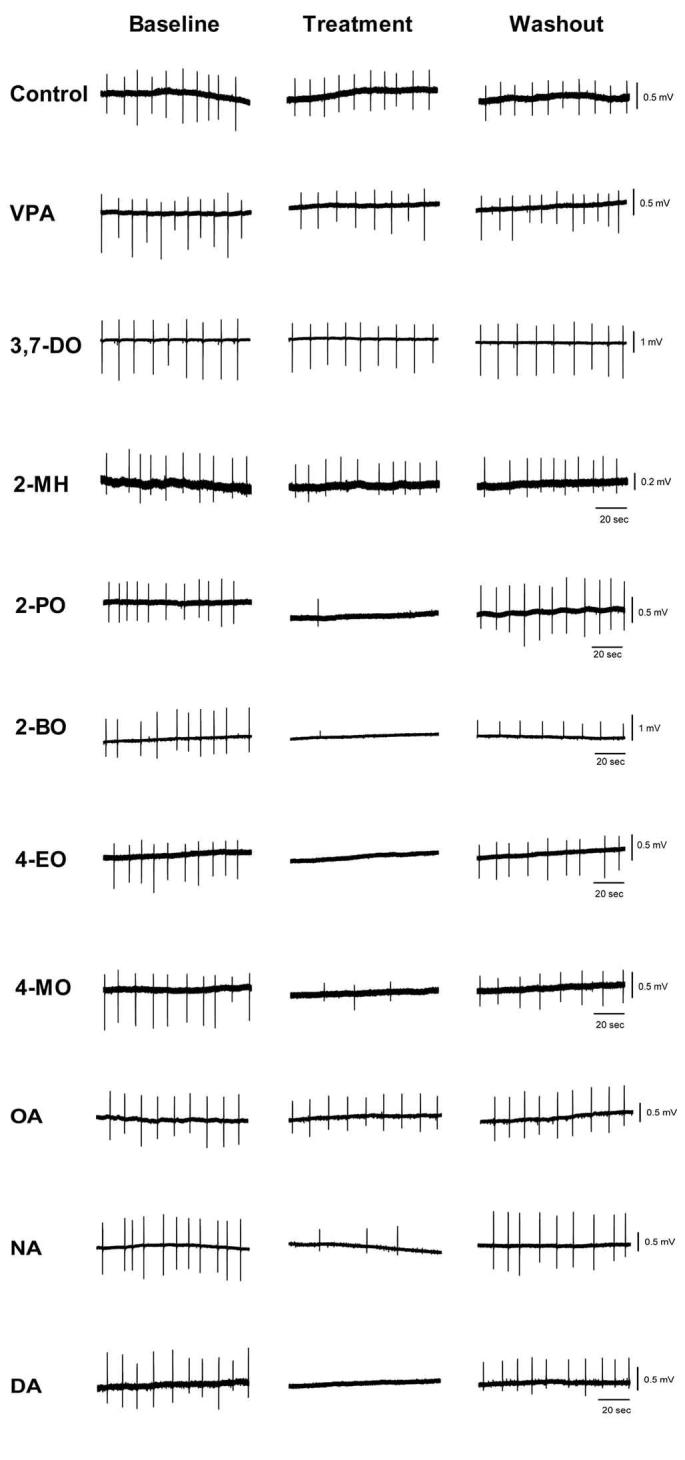
Raw data for spike frequencies for all compounds, as described in Fig. 1.

We have previously identified a range of related medium straight and branched chain fatty acids with a similar mode of action to VPA using the simple model *D*. *discoideum* (Chang et al., 2011; Williams et al., 2006). We thus examined the ability of these compounds to inhibit epileptiform activity in an *in vitro* PTZ model (at 1 mM) in which VPA is only partially effective (Armand et al., 1998), where epileptiform discharges consisted of a positive field potential on which multiple population spikes were superimposed. VPA caused a small but significant decrease in the frequency of epileptiform discharges (77.1 ± 2.0% of baseline) (Fig. 1 and Supplementary Fig. S1). Octanoic acid had no effect on seizure control (OA; 98.4 ± 7.2%), whilst nonanoic and decanoic acid exhibited marked effects (NA; 23.2 ± 8.2% and DA; 2.2 ± 1.4%) (Fig. 1 and Supplementary Fig. S1). Introduction of a side chain to octanoic acid increased suppression of PTZ-induced epileptiform discharges when branched on the second carbon (2-propyloctanoic acid (2-PO; 11.1 ± 4.6%) and 2-butyloctanoic acid (2-BO; 9.2 ± 7.1%)); or the fourth carbon (4-methyloctanoic (4-MO; 50.0 ± 3.8%) and 4-ethyloctanoic (4-EO; 5.8 ± 3.8%)). Epileptiform discharge inhibition was structurally specific, since 3,7-dimethyloctanoic acid (3,7-DO; 96.7 ± 2.6%) and 2-methylheptanoic acid (2-MH; 84.9 ± 2.5%) had limited effect (Fig. 1 and Supplementary Fig. S1). Since octanoic acid and decanoic acid are elevated in plasma of patients undergoing the MCT ketogenic diet (Haidukewych et al., 1982; Sills et al., 1986a, 1986b), our data supports a possible role for decanoic acid and octanoic acid derivatives in the direct therapeutic mechanism of the diet in seizure control.

**Supplementary Fig. 2:**
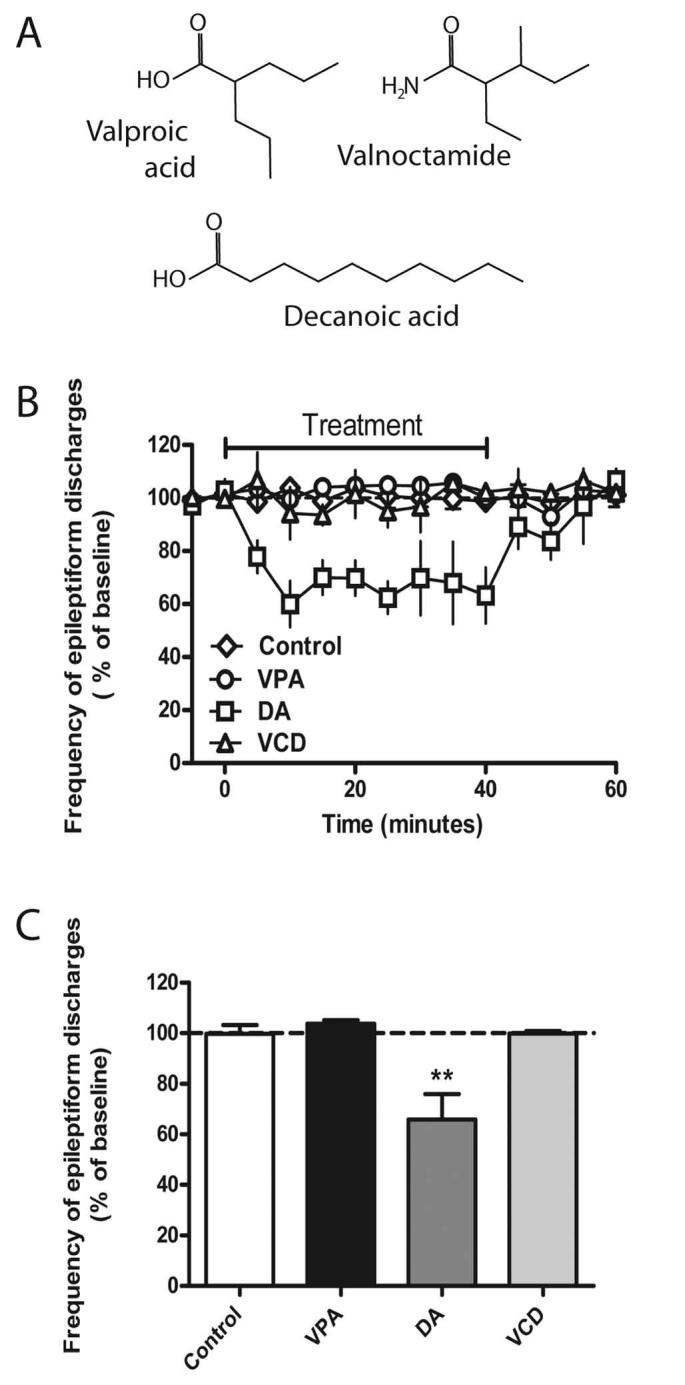
The effect of medium chain fatty acid and related compounds at low concentration on the frequency of PTZ-induced epileptiform discharges in combined entorhinal cortex–hippocampus. (A) Structures of valproic acid (VPA), valnoctamide (VCD) and decanoic acid (DA). (B) The effect of VPA (*n* = 3), DA (*n* = 3) and VCD (*n* = 3) at 0.1 mM on frequency of PTZ-induced epileptiform discharges. The frequency of epileptiform discharges induced by PTZ plotted against time. (B) Comparison of the mean frequency of PTZ-induced epileptiform discharges. Graphs show means ± SEM. ** Indicates a significant difference at *p* < 0.01 compared to control.

In relation to the MCT ketogenic diet, it must be noted here that the concentration of decanoic acid in the serum of patients on the diet (mean concentration 157 μM (Haidukewych et al., 1982)) is lower than that used in our *in vitro* assay. Repeating these epileptiform discharge control experiments using lower decanoic acid concentration (100 μM), we found a significant reduction in the frequency of epileptiform discharges (Supplementary Fig. S2) that was superior to equimolar VPA. In addition, decanoic acid potency was also higher than that of valnoctamide (VCD; see Supplementary Fig. S1 for structure) – a new second generation VPA derivative (Bialer and White, 2010). These data indicate that decanoic acid, at concentrations found in patients during treatment with the ketogenic diet, may provide improved seizure control compared to current and new-generation treatments.

### 3.2 The effect of medium chain fatty acids on human histone deacetylase (HDAC) activity

**Fig. 2:**
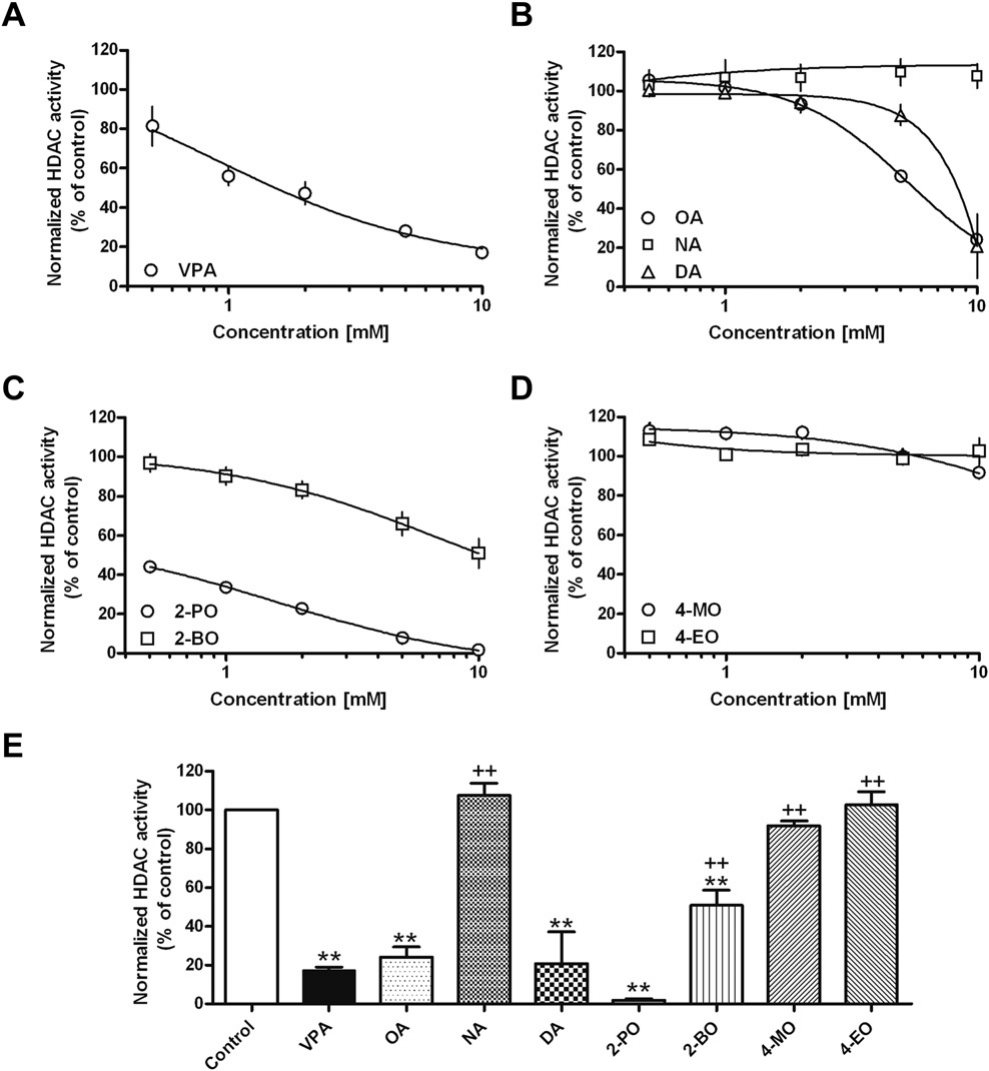
Structurally-specific medium chain fatty acids show reduced inhibition of human histone deacetylase enzyme activity. Quantification of HDAC inhibition assay employing human nuclear extracts enzyme (from HeLa cells) as the source of HDAC activity shown as fitted dose–response curves for (A) VPA; (B) unbranched medium chain fatty acids (OA, NA, DA); (C) medium chain fatty acids branched on the second carbon, 2-BO and 2-PO; and (D) medium chain fatty acids branched on the fourth carbon, 4-MO and 4-EO, showing means ± SEM for four independent measurements at each of five concentrations. (E) Comparison of the HDAC inhibition with different treatments at a concentration of 10 mM, showing means ± SEM for four independent measurements for each compound. ** significant difference at *p* < 0.01 compared to control. ++ significant difference at *p* < 0.01 compared to VPA.

VPA and related fatty acids have been well documented to inhibit HDAC activity (Gottlicher et al., 2001; Gurvich et al., 2004; Phiel et al., 2001), which is likely to cause teratogenicity, and thus limiting their use during pregnancy (Jentink et al., 2010; Koren et al., 2006). We thus screened for this important side effect liability in the compounds showing improved inhibition of epileptiform activity in the PTZ model compared to VPA (Fig. 2). As expected, VPA showed a dose-dependent inhibition of HDAC activity, rising to 82.9 ± 1.8% inhibition at 10 mM. Decanoic acid showed no HDAC inhibition at low concentrations, but a similar inhibitory potency to VPA at 10 mM (79.3 ± 16.5% inhibition). In contrast, nonanoic acid showed minimal inhibitory activity at all concentrations tested (up to 10 mM). As predicted in other studies (Eikel et al., 2006), medium chain fatty acids branched on the second carbon also show high HDAC inhibitory activity (2-propyloctanoic acid and 2-butyloctanoic acid); however, compounds with branching on the fourth carbon did not show HDAC inhibition up to 10 mM. These data show that decanoic acid and compounds branched on the second carbon show undesired HDAC inhibition, whilst this is not evident in related compounds containing a branch on the fourth carbon.

### 3.3 The effect of medium chain fatty acids on hepatotoxicity in a human liver cell line (Huh7)

**Fig. 3:**
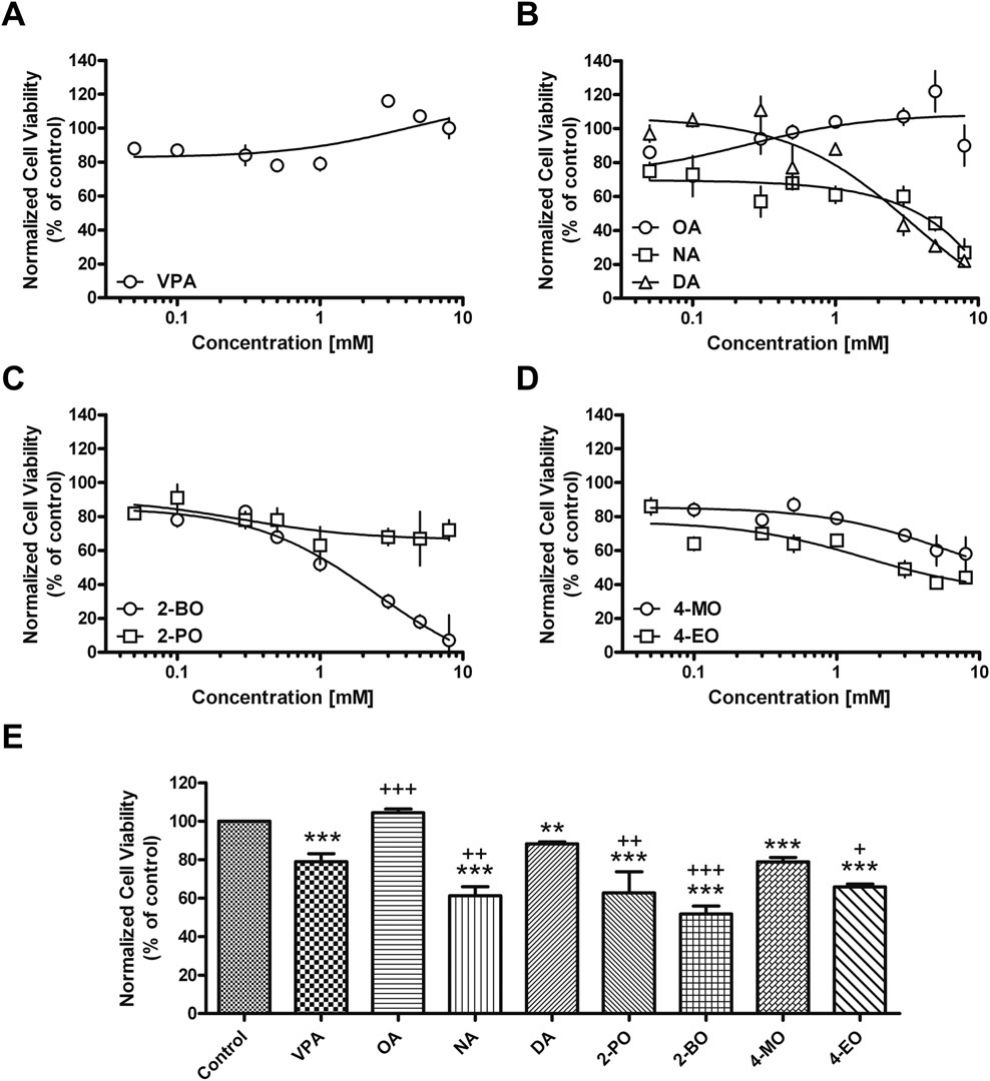
Structurally-specific medium chain fatty acids show variable effects on human liver cell viability. Hepatotoxicity was evaluated by determining the mitochondrial function of human hepatocyte (Huh7) cells following 24 h treatment, measured by the mitochondrial conversion of MTT into blue formazan that decreases in direct proportion to cell viability. Data is provided for (A) VPA; (B) unbranched medium chain fatty acids (OA, NA, DA); (C) medium chain fatty acids branched on the second carbon, 2-BO and 2-PO; and (D) medium chain fatty acids branched on the fourth carbon, 4-MO and 4-EO. Fitted dose–response curves (fitting by three parameters, GraphPad) are based on 8 concentrations and with 4 independent measurements of each concentration. (E) Comparison of hepatotoxicity at a concentration of 1 mM, showing means ± SEM for four independent measurements for each compound. * or + indicate significant difference compared to control or VPA respectively, where one, two or three symbols indicate *p* < 0.5, *p* < 0.1, or *p* < 0.01 respectively.

VPA has also been associated with liver toxicity, possibly through inhibition of β-oxidation (Elphick et al., 2011; Silva et al., 2008). We therefore monitored the effect of the medium chain fatty acids and VPA on hepatotoxicity *in vitro* using human hepatoma cells (Huh7). A decrease in cell number, relative to control (DMSO), was observed at the lowest concentration for all compounds, most likely due to an inhibition of proliferation and not toxicity, consistent with the lack of effect observed using the lactate dehydrogenase assay at these concentrations (data not shown). VPA did not demonstrate any concentration-dependent decrease in viability (Fig. 3), consistent with the finding that VPA-mediated liver toxicity is often underestimated *in vitro* (Nassar et al., 2004). Octanoic acid also demonstrated no significant decrease in viability. Both nonanoic acid and decanoic acid elicited significant concentration-dependent toxicity at concentrations greater than 0.5 mM (IC_50_ = 2.2 mM and 2.9 mM respectively). Branched congeners of octanoic acid did not show effects related to branch position or side chain length, since 4-methyloctanoic acid and 2-propyloctanoic acid showed only minimal toxicity (IC_50_ = 7.3 mM and 9.0 mM respectively), whilst 4-ethyloctanoic acid and 2-butyloctanoic acid were significantly more toxic (IC_50_ = 2.4 mM and 1.1 mM respectively).

Based upon the balance between seizure control and adverse effect liability, 4-methyloctanoic and nonanoic acid were selected for further study.

### 3.4 Dose dependence of 4-methyloctanoic acid and nonanoic acid on *in vitro* epileptiform activity

**Supplementary Fig. 3:**
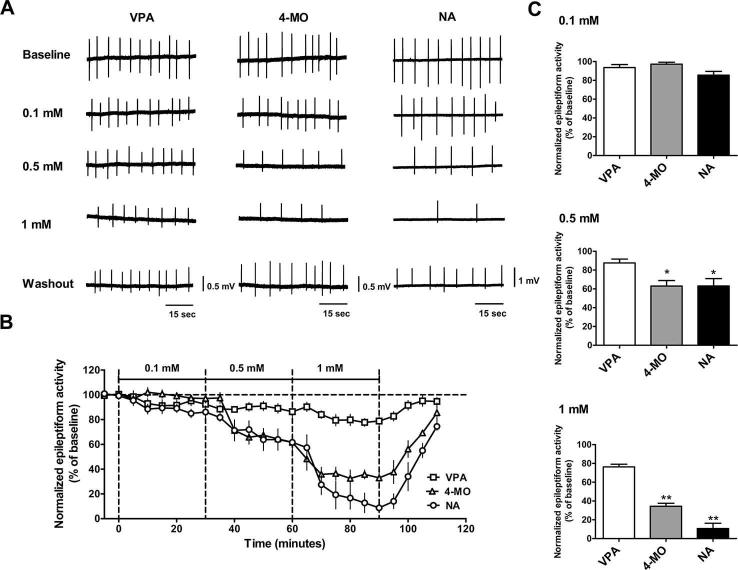
Concentration-dependent *in vitro* seizure control activity for 4-methyloctanoic acid and nonanoic acid compared to VPA. PTZ-induced epileptiform discharges in combined entorhinal cortex–hippocampus was monitored at three compound concentrations. (A) The example of trace record of VPA, 4-MO and NA (B) and frequency of epileptiform discharges plotted against time (*n* = 5 for all). (C) Comparison of the mean frequency of PTZ-induced epileptiform discharges at different treatment concentrations, showing mean ± SEM. *p < 0.05 compared to control, **p < 0.01 compared to control.

We then investigated the dose-dependency of 4-methyloctanoic and nonanoic acid compared to VPA for seizure control in the *in vitro* PTZ model (Supplementary Fig. S3). Both novel fatty acids showed a highly significant improvement in seizure control at 1 mM compared to VPA, and showed improved effectiveness at 0.5 mM. These results suggest that lower concentrations of 4-methyloctanoic acid and nonanoic acid are required to protect against seizures compared to VPA. Importantly, only minimal liability for adverse effects was observed at 0.5 mM, a concentration at which significant seizure control was achieved (see Figs. 2 and 3).

### 3.5 The effect of 4-methyloctanoic acid and nonanoic acid on an *in vivo* electrical stimulation model of status epilepticus

**Fig. 4:**
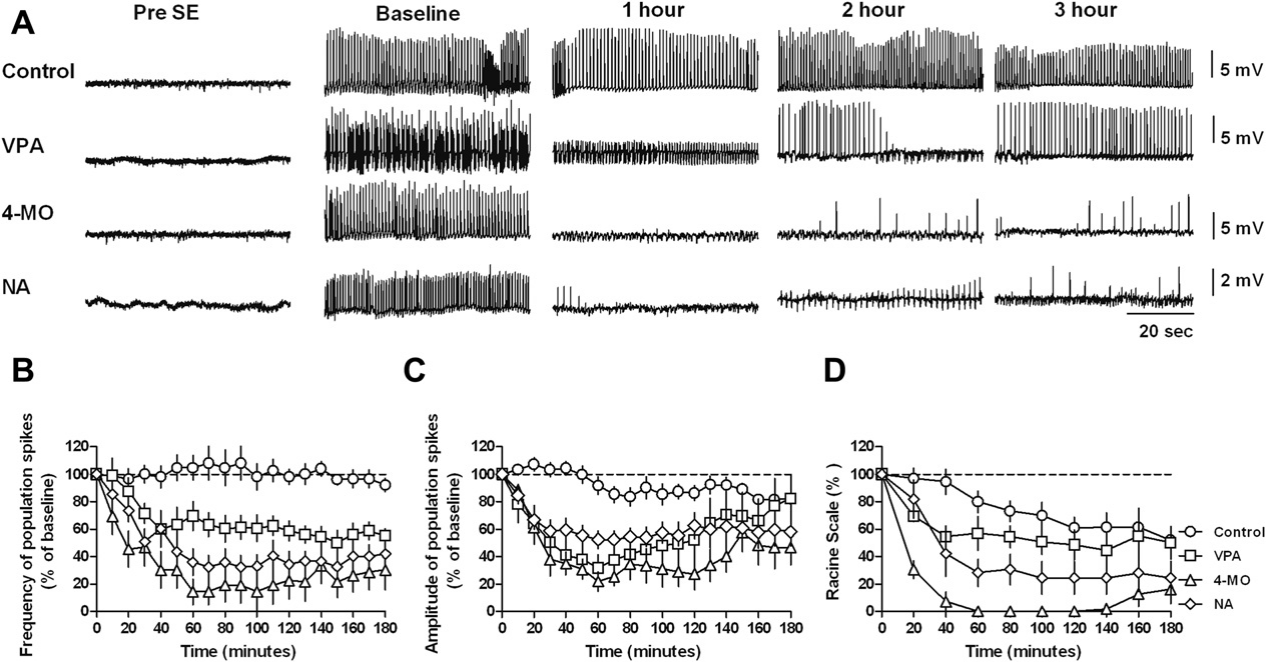
EEG recording spike frequency and amplitude and seizure behaviour following status epilepticus induction is decreased by 4-methyloctanoic and nonanoic acid compared to VPA. (A) Illustrative examples of EEG recordings from animals in self-sustaining status epilepticus (SSSE), induced by perforant pathway stimulation, with traces shown prior to compound administration (baseline) and following administration of control (DMSO), or VPA, 4-MO or NA (all at 400 mg/kg). (B) Time course of the effects on spontaneous spike frequency following administration of control (DMSO; *n* = 5), VPA, 4-MO or NA (*n* = 7 for all at 400 mg/kg) 10 min after stopping perforant path stimulation. (C) Time course of the effects on spontaneous spike amplitude following administration of control (DMSO; *n* = 5), VPA, 4-MO or NA (*n* = 7 for all at 400 mg/kg) 10 min after stopping perforant path stimulation. (D) Time course of the effects of compounds on behaviour seizure which was scored using the Racine score during SSES induced by perforant pathway stimulation.

We next examined the *in vivo* potency of these compounds, using an electrical stimulation model of status epilepticus. In these experiments, perforant path stimulation was used to induce status epilepticus in awake, freely moving rats (Holtkamp et al., 2001; Walker et al., 1999). Administration of vehicle control had no effect on the spike frequency over the 3 h post-stimulation (Fig. 4). VPA (400 mg/kg) decreased the average spike frequency (64.3 ± 10.5%, 60.6 ± 7.8% and 56.6 ± 6.4% of baseline in the first, second and third hours respectively), but did not stop status epilepticus in any animal. In contrast, 4-methyloctanoic acid (400 mg/kg) elicited a much greater decrease (24.9 ± 10.9%, 18.7 ± 13.8% and 28.3 ± 13.0% of baseline in the first, second and third hours respectively), and terminated status epilepticus in all animals. Nonanoic acid was less effective than 4-methyloctanoic acid, but more effective than VPA (46.6 ± 8.1%, 18.7 ± 13.8% and 28.3 ± 13.0% of baseline in the first, second and third hours respectively). Analysis of spike amplitude (Fig. 4A and C) showed a similar improvement in status control.

Control of behavioural responses associated with seizures occurrence was also assessed following continuous perforant path stimulation, ranging from wet dog shakes to increasingly severe tonic-clonic seizures (Fig. 4D). By the end of the stimulation, all animals had reached behavioural seizures characterised as Racine stage 5. In the vehicle control group, animals gradually recovered from status epilepticus (illustrated by a reduction in baseline clinical seizure severity immediately after induction to 80.0 ± 8.0%, 60.8 ± 8.1% and 51.8 ± 5.6% of baseline in the first 3 h respectively). VPA resulted in a non-significant trend for a reduction in seizure severity. In contrast, 4-methyloctanoic acid was more potent than VPA in this model, and led to the complete termination of behavioural seizures in the first 2 h (0.0 ± 0.0, 0.0 ± 0.0% and 18.8 ± 11.8% of baseline in the first 3 h respectively). Nonanoic acid did not terminate behavioural seizures, but showed a highly significant improvement in seizure severity in first hour (28.2 ± 10.8%), and elevated seizure control at later time points compared to VPA (24.4 ± 12.5% of baseline in both the second and third hours). There was also a significant effect of treatment on normalized discharge frequency, discharge amplitude and Racine score (*p* < 0.0001, *p* = 0.005, *p* = 0.001 respectively, repeated measures ANOVA). Post-hoc analysis revealed that compared to control: VPA, 4-methyloctanoic acid and nonanoic all significantly reduced spike frequency (*p* = 0.01, *p* < 0.0001, *p* < 0.001 respectively, Dunnett *t* test) and spike amplitude (*p* < 0.05, *p* = 0.001, *p* < 0.05 respectively, Dunnett *t* test) but only 4-methyloctanoic acid and nonanoic significantly reduced the Racine score (*p* < 0.05, *p* < 0.001 respectively). In comparisons between the treatments 4-methyloctanoic acid had a significantly greater effect on spike frequency and Racine score than did VPA (*p* < 0.05 for both, Tukey HSD). In summary, both novel compounds were far more potent at controlling seizures compared to VPA, with 4-methyloctanoic acid completely terminating behavioural seizures.

### 3.6 The effect of 4-methyloctanoic acid and nonanoic acid on sedation

**Fig. 5:**
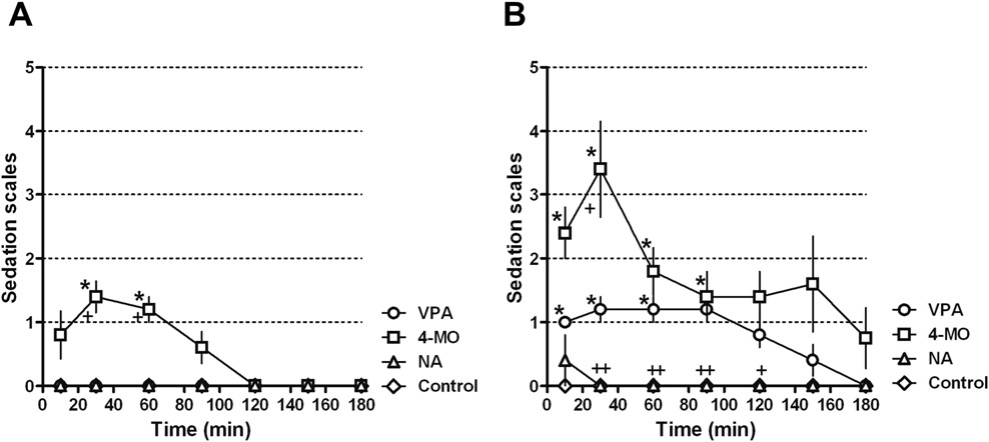
Sedation effects for 4-methyloctanoic and nonanoic acid compared to VPA. Time course of the effect on sedative score following administration of control (DMSO), VPA, 4-MO or NA at (A) 400 mg/kg or (B) 600 mg/kg. All results are obtained from five animals. Sedation score: 0, spontaneous movement; 1, intermittent spontaneous movement; 2, no spontaneous movement; 3, loss of auditory reflex; 4, loss of corneal reflex; 5, loss of response to tail pinch, with data presented as mean ± SEM and significance given by the Mann–Whitney test. *p < 0.05 compared to control; +*p* < 0.05 compared to VPA; **p < 0.01 compared to control; ++*p* < 0.01 compared to VPA.

Neurotoxicological sedation is a typical side effect of most anti-epileptic drugs, especially at high concentrations. In order to compare sedation effects caused by 4-methyloctanoic acid and nonanoic acid to VPA, we first administrated 200 mg/kg of VPA and found no sedation effect (data not shown). Increasing VPA dosage (400 mg/kg) showed no sedative effect (Fig. 5A), in a similar manner to nonanoic acid at this dose, whilst 4-methyloctanoic acid elicited mild sedation – giving a mean sedation score of 1.2 ± 0.2 in first hour and no sedation in both the second and third hours. At higher concentrations (600 mg/kg), VPA elicited a clear range of sedative effects (mean sedation score 1.1 ± 0.1, 1.0 ± 0.0 and 0.2 ± 0.1 in first 3 h respectively), whilst nonanoic acid elicited slight sedation (mean sedation score of 0.1 ± 0.1 in first hour and no sedation in the second and third hours; Fig. 5B). Finally, 4-methyloctanoic was more sedative than VPA at this higher concentration (mean sedation score: 2.5 ± 0.4, 1.4 ± 0.4 and 1.5 ± 0.7 in first 3 h respectively), and one animal died 170 min after administration of the high dose 4-methyloctanoic acid. In summary, 4-methlyoctanoic acid elicits stronger sedative effects whilst nonanoic acid caused a reduced (minimal) sedative effect compared to VPA.

### 3.7 The neuroprotective effect of 4-methyloctanoic acid and nonanoic acid following status epilepticus

**Fig. 6:**
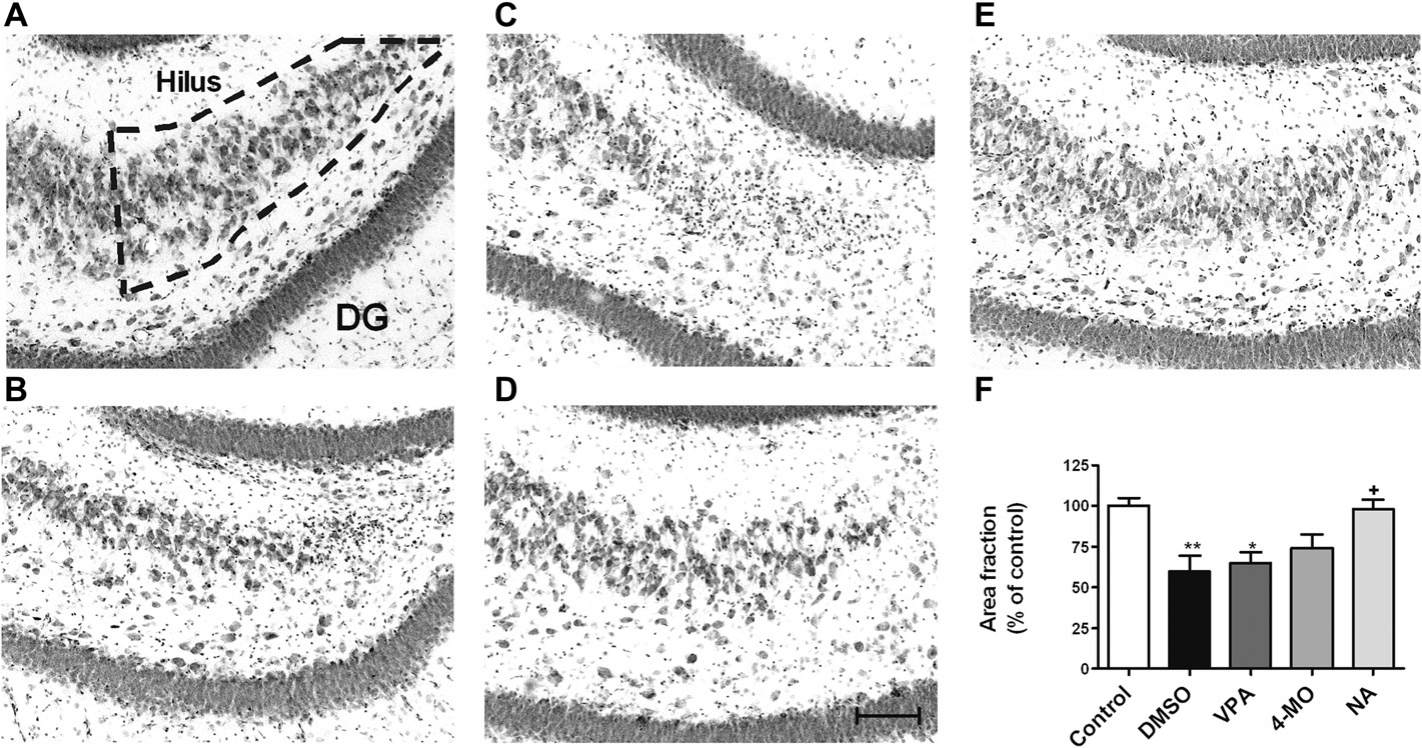
Neuroprotective effects in the hilus of the hippocampus following status epilepticus following nonanoic acid, 4-methyloctanoic acid and VPA treatment. Two months after induction of status epilepticus (following indicated treatments), hippocampal slices were prepared and neuronal cell loss was visualised (illustrated by diffuse dark staining) in the hilus (outlined in A). Animals (A) without status epilepticus (control, *n* = 5) or following status epilepticus that were treated with (B) vehicle only (DMSO) (*n* = 4); (C) VPA (*n* = 6); (D) 4-MO (*n* = 4); and (E) NA (*n* = 3) at 400 mg/kg. (F) Quantification of neuroprotective effect. Graph shows means ± SEM. *p < 0.05 compared to control, **p < 0.01 compared to control; +*p* < 0.05 compared to control. Scale bar = 20 μm. DG; dentate gyrus.

Since our data suggest a potential role for medium chain fatty acids in seizure control, we then extended this study to include possible neuroprotective effects that may provide an active part in the protection against epilepsy. Comparison of neuronal loss following status epilepticus in the absence or presence of treatment enabled a quantification of neuroprotective effects. Following status epilepticus, the hilus was significantly damaged in animals treated with vehicle control (DMSO; 59.8 ± 9.8% of control, *p* = 0.008 compared to no status group) (Fig. 6). Neuronal loss in the hilus was also accompanied by an apparent destruction of CA3 and CA1 pyramidal layers, while the granule cell layer of the dentate gyrus and CA2 showed a resistance to neuron loss after status epilepticus. Application of VPA and 4-methyloctanoic acid were unable to prevent hilar neuronal loss, 65.0 ± 6.5% of control, and 74.4 ± 8.1% of control, respectively, whilst nonanoic acid provided protection (98.2 ± 5.9% of control, *p* = 0.029 and *p* = 0.045 compared to DMSO and VPA respectively; Fig. 6).

## 4 Discussion

The (MCT) ketogenic diet is used extensively for treating refractory childhood epilepsy, although its mechanism of action is poorly understood. As a potential mechanism for this, the medium chain triglycerides given in the diet are largely composed of two unbranched fatty acids (octanoic and decanoic acids), and these are found in elevated levels in the plasma of patients on the diet (Haidukewych et al., 1982; Sills et al., 1986a, 1986b). We have previously demonstrated that both compounds related to these medium chain fatty acids and VPA give rise to similar cellular effects in a simple biomedical model, *Dictyostelium* (Chang et al., 2011; Elphick et al., 2011; Williams et al., 2006, 2002; Xu et al., 2007). In order to develop these compounds for clinical use in epilepsy treatment, they must show greater potency than current anti-epileptic drugs – and preferably in relation to refractory epilepsy – plus fewer side effects and ideally disease modification (Bialer and White, 2010; Galanopoulou et al., 2012). We chose an *in vitro* seizure model that is resistant to a number of antiepileptic drugs (Armand et al., 1998), to select drugs that are more potent than VPA, and then tested these in a model of status epilepticus in which we could test not only an anticonvulsant effect but also a neuroprotective effect.

We show that the straight chain ten carbon (decanoic), but not the eight carbon (octanoic) acid, shows strongly improved seizure control compared to VPA. Our data thus implicates decanoic acid in the seizure control activity of the MCT ketogenic diet. This role is further supported since we show significant seizure control at decanoic acid concentrations correlating with levels shown in the plasma of patients on the diet (Haidukewych et al., 1982). Although our study shows a lack of effect of octanoic acid in seizure control, and this is supported by others (Brill et al., 2006; Hou et al., 2010; Liu and Pollack, 1994; Loscher and Nau, 1985), a recent study has shown octanoic acid increases threshold in the PTZ and 6-HZ seizure test via prior application (Wlaz et al., 2012). Differences between these outcomes may reflect the oral administration of octanoic acid giving rise to metabolism-derived products blocking seizure induction (Wlaz et al., 2012) which were not produced in the other studies (Liu and Pollack, 1994; Loscher and Nau, 1985), or due to a differential response between pre- and post-seizure compound treatment.

Although a role for some medium chain fatty acids (Bojic et al., 1996; Chapman and Meldrum, 1983; Liu and Pollack, 1994; Loscher and Nau, 1985) and fatty acids branching from the second carbon (like VPA) have been demonstrated in seizure control (Bialer and White, 2010; Galanopoulou et al., 2012), this study is the first to identify both nonanoic acid (commonly pelargonic acid) and medium chain fatty acids branching on the fourth carbon as effective seizure control treatments. Both these compounds are not found in MCT oil (Sills et al., 1986a), thus levels of the compounds have not been investigated in the plasma of patients on the diet. However, octanoic acids branched at the fourth carbon are found in lamb fats (Brennand and Lindsay, 1992), at concentrations around 2–5 mg/kg in adipose tissue (Sutherland and Ames, 1994) giving rise to lamb flavour, and sheep and goat milk fat (but not cow) also contain small but significant amounts of 4-methyloctanoic which contributes to mutton- and goat-like flavours in both milk and cheese products (Ha and Lindsay, 1993). Nonanoic acid is also naturally occurring, both in lamb fat at concentrations up to 250 mg/kg (Sutherland and Ames, 1994), and in various plant tissues at concentrations up to 20 mg/kg (Berrie et al., 1975). These concentrations are very low compared to the related octanoic and decanoic acid found at around 6–7% of coconut fat (Beare-Rogers et al., 2001). These orally ingested medium chain fatty acids would be expected to be rapidly taken up in the liver (via the portal vein (St-Onge and Jones, 2002)), and are quickly metabolized via coenzymeA intermediates through β-oxidation and the citric acid cycle to produce carbon dioxide, acetate and ketone bodies (Furman et al., 1965; Huttenlocher et al., 1971; Linscheer et al., 1966). It remains unclear if either the odd number of carbons in nonanoic acid, or the branching in 4-methyloctanoic acid elevates ketone body formation. Our data would suggest, however, that modifying the medium chain fatty acid content of the MCT diet (for example to include nonanoic acid or branched chain compounds) may provide a significant improvement to MCT oil in seizure control.

Continued analysis of 4-methyloctanoic acid and nonanoic acid demonstrated an improved dose-dependence for seizure control compared with VPA, and this translated to a greater effect for these compounds on an *in vivo* model of status epilepticus. *In vitro*, nonanoic acid showed a greater effect than 4-methyloctanoic acid, but this was reversed *in vivo*, presumably due to the lower half-life of straight chain versus branched fatty acids *in vivo* (Chapman and Meldrum, 1983) since it would be very rapidly degraded by first-pass metabolism (Furman et al., 1965; Huttenlocher et al., 1971; Linscheer et al., 1966). The preliminary data presented here suggest that that 4-methyloctanoic acid and nonanoic acid may be more potent than VPA in treating seizures.

The mechanism of seizure control exerted by 4-methyloctanoic acid and nonanoic acid is currently unclear, and speculation on this mechanism must be based upon the mechanism of both the ketogenic diet and VPA. The ketogenic diet has a range of potential therapeutic mechanisms (reviewed in Freeman et al., 2006; Rho and Sankar, 2008), including the production of ketone bodies in the liver (acetone, acetoacetic acid, and β-hydroxybutyric acid) or the regulation of energy metabolism (in the absence of carbohydrates). In our study, where seizure activity was induced in hippocampal slices in circulating, glucose-rich artificial cerebrospinal fluid, it is unclear if sufficient ketone body formation or glucose reduction would occur to control seizures in the rapid time frame shown. Our data instead, suggests a direct mechanism for seizure control in this model by structurally specific fatty acids. It remains to be examined if this mechanism is dependent upon regulation of phosphoinositide signalling, as initially suggested in the discovery of these compounds (Chang et al., 2011).

The teratogenicity of VPA is a major limitation in its use, especially as 25% of people with epilepsy are women of childbearing age (Wallace et al., 1998). VPA teratogenicity has been proposed to occur through HDAC inhibition (Eikel et al., 2006; Gurvich et al., 2004; Phiel et al., 2001), as non-teratogenic analogues of VPA do not inhibit HDACs (Eikel et al., 2006; Phiel et al., 2001). As previously reported (Eikel et al., 2006), compounds branched on the second carbon show high HDAC inhibition. Unexpectedly, the straight chain decanoic acid also showed inhibitory effects at high concentrations, limiting its potential as a new seizure control therapy. However, nonanoic acid and compounds branched on the fourth carbon (4-methyloctanoic and 4-ethyloctanoic acid) exhibited no effect on HDAC inhibition, suggesting that they are unlikely to show HDAC inhibition-related teratogenicity. A subsequent comprehensive analysis of teratogenicity must include *in vivo* studies to examine possible birth defects caused by these compounds in animal models.

VPA is an idiosyncratic hepatotoxin, most likely due to inhibition of fatty acid β-oxidation in the mitochondrial matrix (Silva et al., 2008), increasing the lipid droplet accumulation in hepatocytes (Elphick et al., 2011; Fujimura et al., 2009). Recent studies have shown liver cell toxicology (using a Huh7 cell line) caused by medium chain fatty acids and other VPA-related compounds is dependent on structure features of the compounds (Elphick et al., 2011). Thus, in our current study, we examined the hepatotoxic potential of medium chain fatty acids by determining the mitochondrial function of Huh7 cells using the MTT assay. Interestingly, no common (structurally based) trends were seen for liver cell toxicology in the tested compounds. From the compounds shown to inhibit seizure control *in vitro* above that of VPA, that also lack HDAC inhibitory effects and show minimal effect on liver cell survival (with higher IC_50_ values for hepatotoxicity), 4-methyloctanoic acid and nonanoic acid provide the best profile.

Sedation is the most common side effect of anti-epileptic treatments, including VPA, leading to adverse patient response and often loss of compliance. Our results shows that the sedative effects of medium chain fatty acids are likely to vary dependant on structure, since nonanoic acid shows little sedation, whilst 4-methyloctanoic acid shows relatively strong sedation to that of VPA with an intermediate effect. Through translational medicinal chemistry it should be possible to develop a medium chain fatty acid with increased potency against seizures and low sedative activity compared to VPA.

Neuronal cell death caused by status epilepticus has been shown to be a neuropathological feature of temporal lobe epilepsy, giving rise to enhanced seizure development through hippocampal sclerosis and resultant alterations in physiology (Dam, 1980; Scharfman, 2000). Our study confirms neurodegeneration in the hilus of the dentate gyrus following status epilepticus, as is evident in patients with temporal lobe epilepsy (de Lanerolle et al., 1989) and in equivalent animal models (Buckmaster and Jongen-Relo, 1999). Interestingly, nonanoic acid was more potent than 4-methyloctanoic acid in preventing hilar neuronal death despite having a lesser effect on seizure activity, suggesting a disconnection between these effects. Moreover, these preliminary results point to a potential disease modifying effect of these compounds, as has been also suggested for the ketogenic diet (Gasior et al., 2006).

The results of the present study clearly demonstrate the potential for medium chain fatty acids to provide a highly-improved clinical profile in the development of more effective and safer epilepsy treatments than VPA. These compounds include fatty acids prescribed in the MCT ketogenic diet, shown to be elevated in patients during treatment (Haidukewych et al., 1982; Sills et al., 1986a), and provide novel related structures showing increased potency compared to VPA. We show that a range of structurally specific medium chain fatty acids outperform VPA in seizure control activity, both *in vitro* and *in vivo*, whilst having lower adverse effect profiles. Although we show variable sedative effects, we also show improved neuroprotection (post status). Thus, the family of medium chain fatty acids identified here provides an exciting new field of research with the potential of identifying, stronger, and safer epilepsy treatments.

## Footnotes

Appendix A Supplementary data related to this article can be found at http://dx.doi.org/10.1016/j.neuropharm.2012.11.004.
